# Effect of Structurally Modified Toluene Diisocyanate-Based Polyurethane Pads on Chemical Mechanical Polishing of 4H Silicon Carbide Substrate

**DOI:** 10.3390/polym17050613

**Published:** 2025-02-25

**Authors:** Yiming Meng, Shanduan Zhang, Zefang Zhang

**Affiliations:** 1 Institute for Electric Light Sources, Fudan University, 2005 Songhu Road, Shanghai 200438, China; 2 Shanghai Yingzhi Polishing Materials Co., Ltd., Building 48, Lane 3938, Huqingping Road, Qingpu District, Shanghai 201703, China; zfzhang@fudan.edu.cn; 3 Department of Macromolecular Materials and Engineering, College of Chemistry and Chemical Engineering, Shanghai University of Engineering Science, 333 Longteng Road, Shanghai 201620, China

**Keywords:** microphase separation, toluene diisocyanate-based polyurethane, polycarbonate diol modification, chemical mechanical polishing, silicon carbide substrate

## Abstract

This study investigates the impact of polycarbonate diol (PCDL)-modified toluene diisocyanate (TDI)-based polyester polyurethane polishing pads on the chemical mechanical polishing of 4H silicon carbide (4H-SiC) substrates. Employing a unique metho, PCDL alters the ratio of polyurethane soft and hard segments, facilitating the one-step synthesis of a polishing pad via chemical foaming. The extent of the reaction of isocyanate groups was characterized by Fourier transform infrared spectroscopy, while the changes in the glass transition temperature of the material before and after modification were evaluated using differential scanning calorimetry. The mechanical properties and surface morphology of the modified pad have been systematically characterized. The results showed that compared with the polyurethane polishing pad without PCDL, tensile strength was augmented by a factor of 2.1, the elastic modulus surged by a factor of 4.2, the elongation at break improved by a factor 1.6, and the wear index decreased by a factor of 0.5 by 40 wt.% PCDL loading. Furthermore, the modified pad demonstrated a 14.5% increase in material removal rate and a reduction in surface roughness of 4H-SiC from 0.124 nm to 0.067 nm. Additionally, the compact surface pore structure and enhanced chemical stability in the strong oxidizing slurry of the modified pad enabled superior polishing performance, achieving an ultrasmooth 4H-SiC surface. The study highlights the potential of tailored polyurethane formulations in enhancing polishing efficiency and surface finish in semiconductor manufacturing processes.

## 1. Introduction

4H silicon carbide (4H-SiC) shows great potential as a third-generation high-frequency and high-power semiconductor material that has garnered significant attention both currently and for the foreseeable future [1,2]. Its prominence in semiconductor applications stems from its exceptional performance in withstanding high temperatures and frequencies [3,4,5], making it a sought-after choice. Typically, 4H-SiC finds primary utilization in device epitaxy, where the quality of the substrate surface critically influences the quality of both homogeneous and heterogeneous epitaxy [6], as well as the longevity of the devices. Thus, it holds a pivotal position in the realm of electronic integrated manufacturing [7]. Despite its inherent physical characteristics, including a Mohs hardness of 9.4 and remarkable chemical stability, marked by a decomposition temperature of 1700 K, a melting point of 3100 K for 4H-SiC, and an even higher melting point of 4000 K for carbon [8,9,10], the machining of 4H-SiC presents a formidable challenge. Its unique material properties render it difficult to achieve both a high removal rate and an impeccable surface finish. Especially, in the CMP processing of the SiC substrate, for which surface roughness is highly demanded, the low removal rate is the main problem [11,12,13].

A CMP process could be significantly influenced by many factors, such as the pad, slurry, rotation speed, down force, and slurry flow rate, etc. Among these, the polishing pad assumes a crucial function [14,15,16,17,18,19]. Polyurethane (PU) pads, renowned for their exceptional durability and longevity, have been extensively utilized for polishing SiC substrates. The elasticity, waterproof nature, and high chemical resistance of PU elastomers make them an ideal choice as a matrix material for polishing pads, contributing to the precision and effectiveness of the CMP process. The performance of PU polishing pads is mainly determined by the following three factors: porosity, cellular microstructure, and polymer properties [20]. Chemically, the pad must be able to withstand aggressive slurry chemistries used in CMP polishing without degrading, delaminating, blistering, or warping. More specifically, the strong oxidizing KMnO_4_-based slurries, which can be either highly alkaline (near pH 11) or highly acidic (near pH 2), can improve SiC polishing efficiency [21,22,23]. These conditions impose stringent requirements on the chemical resistance and mechanical strength of the polymer matrix, ensuring the pad can endure rigorous polishing conditions, including exposure to aggressive chemicals and high mechanical stress. The chemical structure and functionality of polyols and isocyanates significantly influence the properties of polyurethanes. When polyether polyol is used as soft segments, the PU will have excellent flexibility and low temperature properties. However, the PU still shows defects in mechanical properties [24,25,26]. Efforts to enhance the mechanical properties and water resistance of polyether-based polyurethanes while maintaining their desirable molecular weights and optical properties have been intensive. One effective approach involves incorporating a blend of soft segments, consisting of both polyether polyols and polycarbonate polyols [27,28,29,30,31]. However, other materials used in polyurethanes can also influence properties such as density, porosity, morphology, mechanical strength, and the rate of reaction. Material properties are also affected by other factors such as catalysts, chain extenders, cross-linkers, surfactants, and blowing agents. Few studies explore the influence of polycarbonate diol (PCDL)-modified toluene diisocyanate (TDI)-based polyether polyurethane materials on the development of chemical resistance in the polishing process of SiC substrates under strong oxidizing conditions. This includes utilizing water as the blowing agent while simultaneously achieving a balanced relationship between foaming and gelling through the optimal ratio of catalysts, chain extenders, cross-linkers, and surfactants.

In this work, a ratio of 0–40 wt.% PCDL with a molecular weight of 1000 was employed to optimize the soft segment of a TDI-based polyether prepolymer, facilitating the one-step synthesis of a CMP polishing pad tailored for SiC substrates via water foaming. By strategically adjusting the ratio of soft to hard segments, we achieved a polymer matrix with reduced microphase separation, resulting in an increased glass transition temperature. Consequently, significant enhancements were observed in the hardness, elastic modulus, elongation at break, and wear resistance of the composite pad. High-efficiency polishing in the CMP of SiC can be achieved by pads with reinforced bulk materials. Additionally, the dense surface pore structure, superior elasticity, and enhanced chemical stability in strong oxidizing slurry of the pad contribute to a higher polishing efficiency, prolonged lifetime, and superior surface quality of the SiC substrates when employed in a KMnO_4_-based slurry containing MnO_2_ abrasives during the chemical mechanical polishing process. It is possible to obtain a high removal rate and ultra-smooth SiC surface that exceeds that of a dedicated pad. These findings suggest that this approach could provide a valuable alternative for high-performance CMP applications.

## 2. Experimental Section

### 2.1. Materials

The TDI-terminated polyether prepolymer (NCO weight: 9.25–9.65%, LU-T75D) was provided by Covestro Co., Ltd., Shanghai, China. Polycarbonate diol (PCDL, M.wt. = 1000, ETERNACOLL^®®^UH-100) was provided by UBE Corporation, Ube, Japan; 4,4′-Methylenebis-2-chloroaniline (MOCA) was supplied by Suzhou Xiangyuan New Materials Co., Ltd., Suzhou, China. Silicone surfactant (NIAX*L-1500) and 33% Triethylene diamine solution (NIAX*A33) were purchased by Momentive Performance Materials Inc., Niskayuna, NY, USA. COPOL-431 slurry (pH = 2.7) was supplied by Shanghai Yingzhi Polishing Materials Co., Ltd., Shanghai, China. All reagents were used without further purification. The commercial pad (HF2) was purchased from FUJIBO Ehime Co., Ltd., Tokyo, Japan.

### 2.2. Preparation of the Polyurethane Polishing Pad

**Figure 1:**
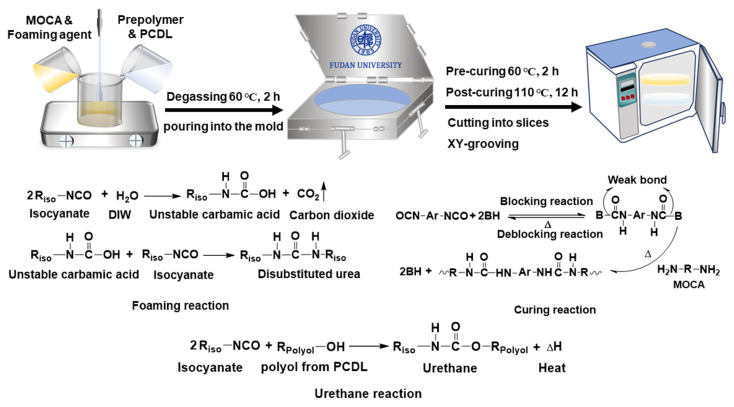
Schematic of the fabrication and reaction of PU/PCDL polishing pads.

The sample was prepared by using the one-step chemical foaming casting technique. Following the typical procedure, PCDL was blended into the polyether prepolymer according to a specific ratio (prepolymer: PCDL = 100:80) with strong mechanical stirring, and the polyblend was degassed in a vacuum oven at 60 °C for 1 h. Then, a foaming solution (L-1500:A33:deionized water = 1:0.03:0.035) and preheated MOCA liquid at 120 °C were added to the mixture at a specific ratio (prepolymer: MOCA = 100:11). Finally, the mixture was poured into a mold and pre-cured at 60 °C for 2 h; then, it was post-cured at 110 °C for 12 h to obtain the foamed PU/40 wt.% PCDL composite. The formulation of the samples is given in Table S1. The PU/PCDL composites were designated as PUPCDx, where PU, PCD, and x represent polyurethane, polycarbonate diol, and the weight percentage of polycarbonate diol in the composite, respectively. It is worth mentioning that the control sample without the addition of PCDL was named Neat PU. For comparison, samples including commercial pad HF2, and composites Neat PU, PUPCD20, and PUPCD40 were prepared using a similar casting technique. The samples were sliced into sheets with a diameter of 915 mm and a thickness of 1.8 mm, and the samples were obtained by grooving the slices with 9 × 9 mm^2^ grids. A schematic of the fabrication process and reaction PU/PCDL polishing pads are shown in Figure 1.

### 2.3. CMP Experiment of SiC Substrate

The CMP polishing process used a 36B single-side polishing machine, with a fixed plate diameter of 914 mm (Zhejiang Morinaga Optical & Electronic Equipment Co., Ltd., Jiaxing, China). Commercial 6-inch N-type single-crystal SiC substrates were used in the present study, and the Si-polar surface of N-type 4H-SiC substrate was the processing surface. Four N-type SiC substrates with a thickness of 440 ± 5 μm and a surface roughness (Ra) of 0.410 ± 0.005 nm were selected for each CMP batch. The acidic COPOL-431 slurry consists of 20 nm MnO_2_, 5 wt.% KMnO_4_, and HNO_3_, controlling the pH value for all polishing tests, provided by Shanghai Yingzhi Polishing Materials Co., Ltd., Shanghai, China. The polished SiC substrates were washed in an ultrasonic bath, using a cleaning solution containing 1 wt.% citric acid in DI water, and they were then cleaned in ethanol three times, blown dry, and subsequently tested by AFM. For each SiC substrate, AFM measurements were taken at the following three points: the upper edge, the center point, and the lower edge. The arithmetic mean of these measurements was calculated to obtain the average Ra. The CMP process parameters of the SiC substrates are shown in Table 1.

### 2.4. Measurement and Characterization

An optical microscope (OM, Axiolab 5, ZEISS Co., Shanghai, China) was used to observe the morphology of the polishing pad. Fourier transform infrared (FT-IR) spectra were recorded using an FT-IR (Nicolet iS20, Thermo Fisher Scientific Inc., Carlsbad, CA, USA) and an attenuated total reflectance (ATR) accessory in the range of 4000–400 cm^−1^ at room temperature. Elemental mappings of the samples were obtained through the EDS detector attached to the Field Emission Scanning Electron Microscope (SEM, ZEISS Sigma 300, Jena, Germany). The tension test was performed according to ASTM D412-16 [32] by a microcomputer-controlled electronic universal material testing machine (STM-S1.05, SITEMA INDUSTRY CO., Ltd., Shanghai, China). The specimens of the pad were prepared in a dumbbell shape, the rate of grip separation was set to 500 ± 50 mm/min, and all the data were the average of 4 individual tension tests. A shore durometer was used to test the hardness of the polishing pad. The surface topography and mean roughness of the silicon carbide were measured using a Dimension Edge System atomic force microscope (AFM, Bruker Corporation, Billerica, MA, USA). The density of the samples was measured by a density balance (resolution: 0.001 g, Shanghai Yue Ping Scientific Instrument Co., Ltd., Shanghai, China). The thickness, compressibility, and recovery of the polishing pads were measured by a ZY-9002-B thickness gauge (Dongguan Zhuoyue Equipment Co., Ltd. and KTS Equipment Technology Co., Ltd., Dongguan, China). The porosity and average pore size of the polishing pads were analyzed by ImageJ version 1.53t 24 August 2022 software. The weight of the silicon carbide before and after polishing was measured by a professional electron balance (resolution: 0.0001 g; Aohaosi Instrument Co., Ltd., Shanghai, China) to calculate the material removal rate (MRR) according to Equation (1).

(1) $$MRR=\frac{10^{4}\times \Delta m}{\rho \times S\times t}$$

Here, Δm (g) is the weight variation of the 6-inch silicon carbide before and after polishing, S (cm^2^) is the 6-inch silicon carbide substrate area, t (h) is the polishing time, ρ is the density of silicon carbide (3.21 g/cm^3^), and MRR (μm/h) is the corresponding material removal rate. All the data were the average of 3 individual polishing tests. The abrasion test was performed according to ASTM-D4060-19 [33] by a ZY-6004-T Taber abrasion testing machine (Dongguan Zhuoyue Equipment Co., Ltd. and KTS Equipment Technology Co., Ltd., Dongguan, China), fixing the sample (A circle with a diameter of 108 mm) on a tested turntable while selecting the H-18 grinding wheel. The experiment was carried out at a speed of 72 rpm and load of 1000 g. The total wear was 1000 r, the weight of the test sample was measures to the nearest 0.1 mg, and the wear index (*I*) [34,35] was calculated after 1000 r according to Equation (2).

(2) $$I=\frac{\Delta m\times 1000}{C}$$

Δm (mg) is the weight variation in the test sample before and after abrasion, C is the number of the cycles of abrasion recorded. All the data were the average of 3 individual abrasion tests.

## 3. Results and Discussion

### 3.1. FT-IR Analysis of the Pad

**Figure 2:**
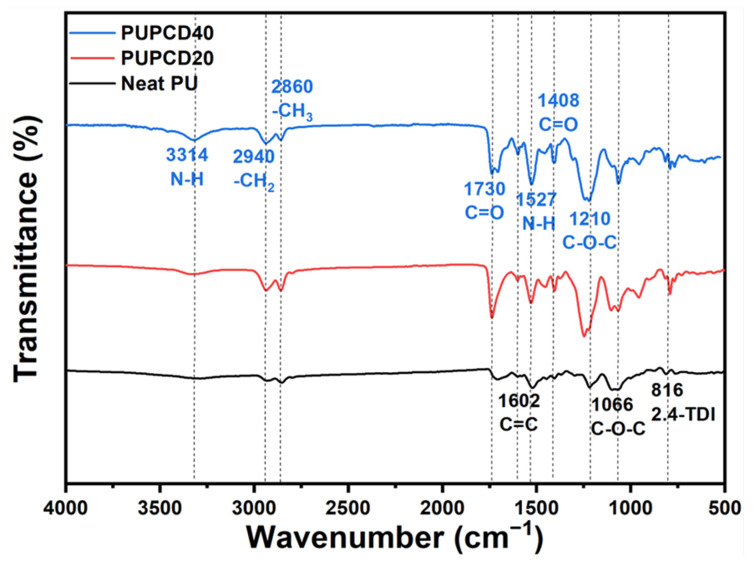
FT-IR spectrum of Neat PU, PUPCD20, and PUPCD40.

FT-IR analysis was conducted on Neat PU, PUPCD20, and PUPCD40, respectively, and this spectral information is presented in Figure 2. This analysis holds significant importance in detecting the functional groups present in the PU-based samples and identifying the differences between the functional groups of PU with various [NCO/OH] ratios and the isocyanate end group of urethane prepolymer [36]. Hydrogen bonds are primarily formed between the polar carbonyl groups and the amino groups of the carbamate groups. In addition to the compatibility between the soft and hard segments, the content and strength of hydrogen bonds serve as critical driving forces for microphase separation in rigid PU foams [37]. The characterization of hydrogen bond content and types, as obtained from the FTIR spectroscopy analysis, is a fundamental method for investigating the microphase separation structure in rigid PU foams [38]. Specifically, the N-H group, acting as the hydrogen donor in the carbamate, can interact with the ether bond (C-O) in the soft segments, as well as the carbonyl group (C=O···H-N) or ether oxygen (C-O···H-N) in the carbamate, to form hydrogen bonds [39].

The disappearance of the vibration band at 2270 cm^−1^ is attributed to NCO groups, indicating that the initial isocyanate groups completely reacted during the synthesis [40]. In the N-H stretching region, the major absorption around 3314 cm^−1^ was attributed to the bonded N-H vibration [41]. This result suggests that a complex morphology develops as the PCDL content increases, involving both the PCDL or PTMG soft segments and the hard segments [42]. For the soft segment polyols, the peak groups 2940 cm^−1^ and 2860 cm^−1^ are caused by the stretching vibration of C-H_2_ and C-H_3_, respectively. The sharp absorption peak is around 1730 cm^−1^, which is due to the stretching vibration of esters C=O [43]. According to the data on PUPCD40 and PUPCD20, the intensity of this peak was enhanced after the addition of PCDL. The absorption peak around 1602 cm^−1^ is a stretching vibration from C=C in benzene ring. The medium-strong peak at 1527 cm^−1^ confirms the in-plane bending vibration of N–H. The peaks around 1710 cm^−1^ and 1408 cm^−1^ correspond to C=O in isocyanurate from TDI. The absorption peak around 816 cm^−1^ is the characteristic peak of the 2,4-TDI benzene ring, C–O–C stretching at 1210 cm^−1^, and 1066 cm^−1^ from the ether [44].

The incorporation of PCDL significantly influences the soft-to-hard segment ratio in the polyurethane matrix. The flexible polyol structure of PCDL increases the proportion of soft segments, enhancing the material’s elasticity and toughness. This change promotes microphase separation, as evidenced by the DSC measurement, which shows an increase in PCDL content from 0% to 40%, which resulted in a corresponding rise in the glass transition temperature (T_g_), from 238.6 °C to 250.8 °C, as depicted in Figure S1. This observation indicates a reduction in the degree of microphase separation [45]. The enhanced phase mixing can be attributed to the reduced stiffness differential between the soft and hard segment chains, which arises from the higher stiffness of PCDL compared to the original PTMG chains. Additionally, the mobility of the soft segments at the hard/soft segment junction may be constrained due to the superior crystallization capacity of the PCDL segments in comparison to the original PTMG segments [46]. Moreover, the stronger interactions between the hard segments and the carbonate groups in PCDL, in contrast to the ether groups in PTMG, may further contribute to this phenomenon. Consequently, the interaction between the hard and soft segments improves the miscibility of the domains, resulting in an increase in T_g_.

With an increase in PCDL content, the ratio of the hard-to-soft segments of the polyurethane material itself changes in the polishing pad [47]. This improved the mechanical properties of the intrinsic properties of polyurethane due to the excellent stiffness of the polycarbonate bonds in PCDL, which could help maintain structural stability [48].

### 3.2. Physical Properties of the Pad

Polyurethane pads are condensation polymers prepared by the reaction of isocyanate and a polyol in the presence of a catalyst and a foaming agent. The molecular structure of isocyanates and polyols significantly influences their inherent polymer properties, such as glass transition temperature, and mechanical properties such as elastic modulus, tensile strength, and elongation at break. The final pads of the mechanical and functional properties of foams are inherently dependent on both the intrinsic properties of the constituent polymer and intricate foam structural attributes, including foam density, cell type (open or closed), and cell morphology [49].

The influence of PCDL on the soft segment structure of polyurethane has been discussed above. Next, we delve into the impact of its chemical structure modification on the physical properties of polishing pads and their relationship with mechanical friction in SiC planarization, especially the parameters of hardness, compressibility, porosity, and wear index. The non-grooved sample Neat PU, PUPCD20, PUPCD40, and HF2 pads are cut into four 10 × 10 cm^2^ squares for the density, hardness, compressibility, porosity measurements, a circle with a diameter of 108 mm for the wear index, and a dumbbell shape for the tension test; the results are illustrated in Table 2. As can be seen from Table 2, with the addition of a modifying agent, PCDL, the density, hardness, and resilience of the material are increased, and the average pore size, compressibility, and porosity are decreased. It is worth noting that the resilience of the polishing pads paradoxically increases with the augmentation of their density. This is different from the high density and low resilience of commercial polishing pad HF2.

At the same time, a mechanical property improvement is concurrently observed in the enhancements of the tensile strength, elastic modulus, and elongation at break, with a reduction in the wear index. This phenomenon may stem from PCDL altering the ratio of hard-to-soft segments, where the hard segment content is identical, and the soft segment has a larger molecular weight, longer length, and more hydrogen bonding contacts between the hard domains [50].

**Figure 3:**
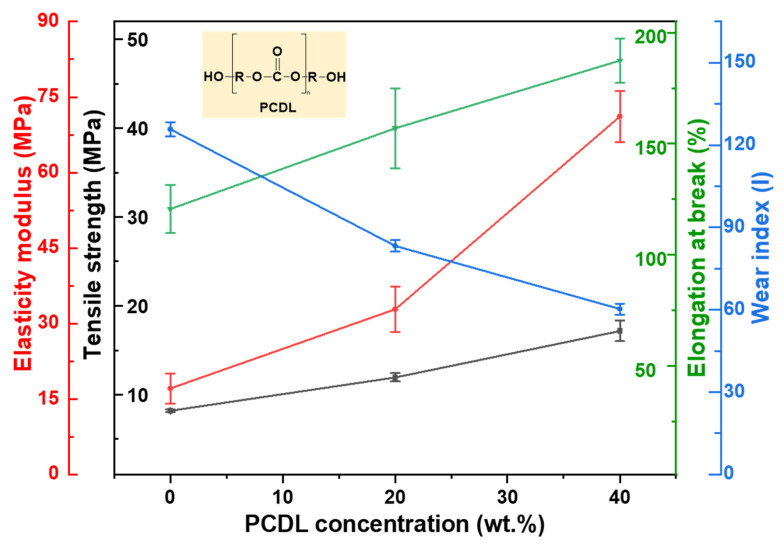
Tension test performance and wear index of polishing pads with different PCDL contents.

Moreover, the addition of PCDL to the improved original soft segment structure makes the phenomenon of microphase separation between soft and hard segments less pronounced. This is attributed to the excellent toughness of the polycarbonate bonds in PCDL, which helps maintain the stability of the structure [48,51]. Consequently, the Neat PU, PUPCD20, and PUPCD40 exhibit a gradual increase in tensile strength, elastic modulus, and elongation at break as the PCDL content increases. Consequently, the tensile strength, elastic modulus, and elongation at break of Neat PU, PUPCD20, and PUPCD40 gradually increased with an increase in the PCDL content, as illustrated in Figure 3.

The aforementioned physical property results demonstrate that increasing the PCDL content from 0 to 40 wt.% leads to substantial enhancements in the bulk material characteristics of the polishing pad samples, including hardness, density, elastic modulus, wear resistance, tensile strength, and elongation at break. In the subsequent phase of this study, we will investigate the variations in the surface morphology of these polishing pad samples, as well as the bulk pore structure, alongside the degree of glazing before and after the CMP process.

### 3.3. Morphological Properties and Chemical Resistance of the Pad

The pore quality, surface quality, chemical resistance, and wear degree of the polishing pad are important factors for evaluating the polishing efficiency and service lifetime of the polishing pad for the SiC substrate. Therefore, the assessment of pad surface and bulk morphology is conducted through optical microscopy (OM).

**Figure 4:**
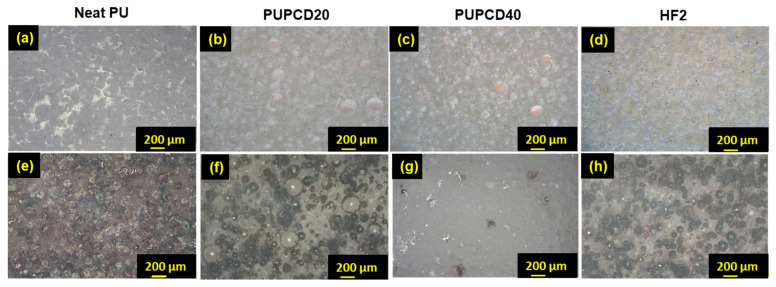
The optical micrographs of (**a**,**e**) Neat PU, (**b**,**f**) PUPCD20, (**c**,**g**) PUPCD40, and (**d**,**h**) HF2 pad surface comparison before and after the 12 h CMP process at the same magnification, respectively.

As shown in Figure 4, with an increase in PCDL content, the number of small pores increases, accompanied by a reduction in the average pore size. This trend corresponds to the results shown in Table 2, indicating that higher PCDL content promotes the formation of a more interconnected pore network while reducing the average pore size, which may enhance the pad’s mechanical and polishing properties. Simultaneously, as the PCDL content increases, both the degree of surface abrasion and the chemical corrosion of the polished polishing pad show improvement. This improvement is especially noticeable when the PCDL content reaches 40 wt.% (Figure 4c,g), showing the most distinct differences in surface morphology compared to commercial HF2 (Figure 4d,h), PUPCD20 (Figure 4b,f), and Neat PU (Figure 4a,e). It retains a more intact pore structure and fewer chemically corroded areas, along with the deposition of MnO_2_ abrasives in the pores (black areas) [21].

**Figure 5:**
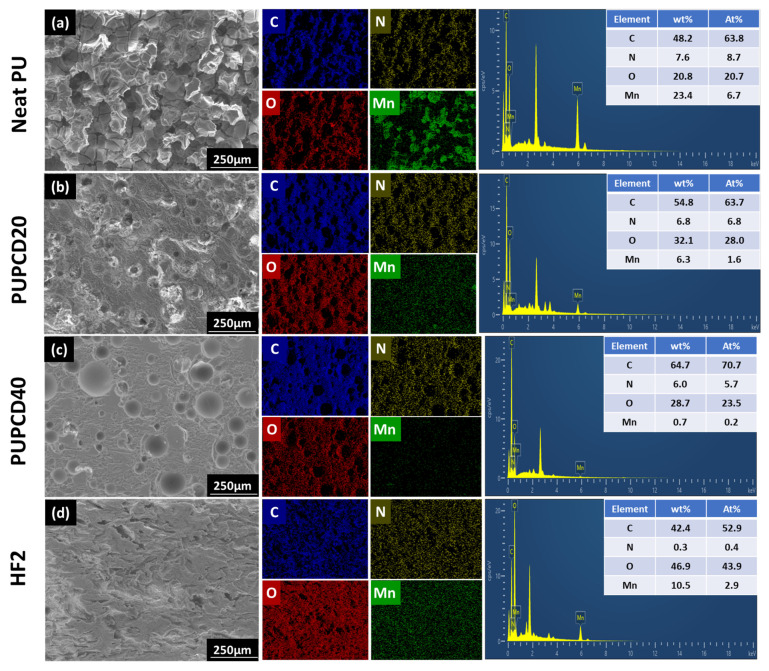
SEM image and the corresponding EDS elemental mapping images and analysis of the polished sample surface of (**a**) Neat PU, (**b**) PUPCD20, (**c**) PUPCD40, and (**d**) HF2, respectively.

As the EDS analysis illustrates in Figure 5, the Neat PU contains the highest content of Mn, predominantly aggregated within pores on the sample surface, leading to the deterioration of its surface structure. However, the PUPCD20 and PUPCD40 samples demonstrate a gradual reduction in Mn distribution across their surfaces, coupled with an increase in the preservation of intact pore morphologies. This observation is consistent with the OM results presented in Figure 4. In contrast, the pore structure of the commercial polishing pad HF2 is disrupted, and its Mn content is higher than that of the samples prepared through PCDL modification. The EDX analysis reveals changes in the content of Mn, as well as other key elements such as C, N, and O. The distribution of carbon is consistent with the organic components of the polishing pad, as shown in the mapping images, indicating material homogeneity. Nitrogen, primarily associated with the urethane groups, is uniformly distributed, aligning with the expected chemical structure of the pad. Oxygen is linked to both the polyurethane matrix and the MnO_2_ particles, with localized regions of higher oxygen content corresponding to the presence of MnO_2_ particles. These findings are supported by the elemental mapping images, which provide a visual representation of the spatial distribution of each element.

This improvement can be attributed to the modification of the soft-to-hard segment ratio of the original prepolymer due to the incorporation of PCDL, which enhances the hydrolytic stability, thermal resistance, and solvent resistance of the original polyether-based prepolymer [52]. Additionally, this modification results in the higher elastic modulus and improved wear resistance of the polishing pad. As the PCDL content in the samples increases from 0 to 40 wt.%, a marked improvement in the degree of abrasive deposition is observed, accompanied by a more cohesive pore structure. This enhancement can be attributed to the stronger hydrogen bonding of soft and hard segment interactions facilitated by PCDL, which significantly enhances the chemical and mechanical stability of the material’s surface through modification [53].

From the surface morphology of the polishing pad before and after CMP, it is observed that PUPCD40 exhibits a well-defined cavity shape, a more complete pore structure, less wear, and fewer particle (abrasives and debris) accumulations. This is expected to facilitate the storage and flow of the polishing slurry on the SiC substrate, leading to superior CMP performance. This observation is further confirmed in the subsequent CMP performance evaluations.

**Figure 6:**
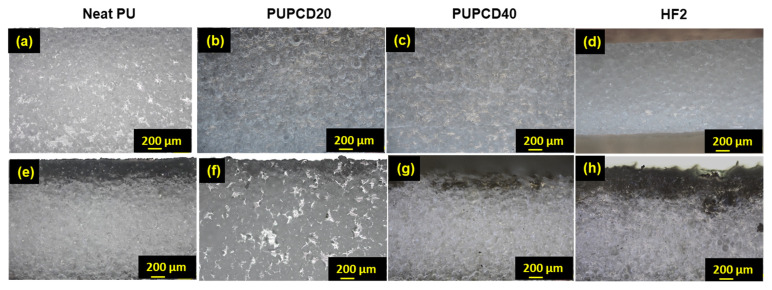
Optical micrographs of the (**a**,**e**) Neat PU, (**b**,**f**) PUPCD20, (**c**,**g**) PUPCD40, and (**d**,**h**) HF2 cross-sectional comparison before and after the 12 h CMP process at the same magnification, respectively.

The cross-sectional morphology of the polishing pad shown in Figure 6 reveals the changes in depth of particle (abrasives, debris) immersion and chemical erosion (the dark regions) before and after the CMP process. Notably, PUPCD40 demonstrates the most favorable condition, exhibiting the shallowest dark region depth. Furthermore, an increase in PCDL content correlates with a trend toward shallower depths of the dark regions. This phenomenon can be attributed to the modification of the soft segment structure by PCDL, which enhances the concentration of hydrogen bonding sites between the hard and soft blocks [52]. Consequently, this leads to improvements in the polishing pad’s hardness, elongation at break, elastic modulus, and variation in porosity. Ultimately, these factors significantly affect the service life and polishing efficiency of the polishing pad, which will be explored in greater detail in the following section on the CMP performance of the polishing pad.

### 3.4. CMP Performance

To better understand the effect of PCDL-modified pad properties on the polishing performance, four different pads were used. The polishing characteristics of these pads were studied under the same polishing conditions of applied pressure, rotation speed, slurry type, slurry flow rates, initial pad conditioning, and polishing temperatures.

**Figure 7:**
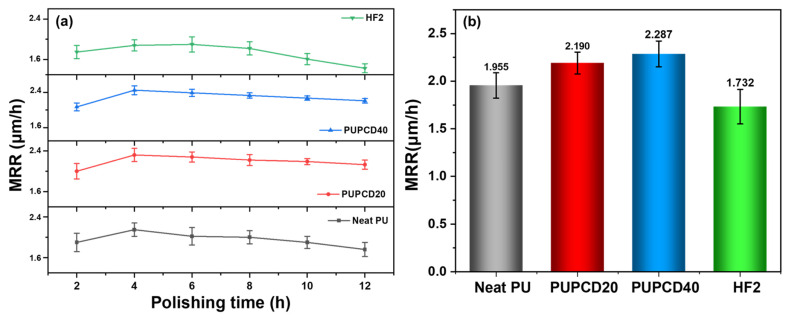
CMP process of each polishing pad’s (**a**) material removal rate and (**b**) average material removal rate within 12 h.

Figure 7 depicts the average material removal rate (MRR) performance of four types of polishing pads during a 12 h continuous polishing of SiC substrates on the Si surface in a high-concentration KMnO_4_-based slurry, as well as the MRR variation trends for each polishing cycle. From Figure 7a, it is evident that with an increase in PCDL content, there is a corresponding rise in MRR for each cycle. The MRR trend is optimal at a PCDL content of 40 wt.%, with a decay rate of only 5.5%, unlike Neat PU (8.1%) and HF2 (18.3%), which show a noticeable MRR decline starting from the third run. This improvement is attributed to the structural modification of polyurethane materials by PCDL, reducing the occurrence of microphase separation and forming a more stable cross-linked structure [48]. As a result, the polishing pads exhibit increased resistance to chemical solvent corrosion, extended service life, and better rate performance. It is noteworthy that all polishing pads demonstrated lower MRR during the first run, which can be attributed to the inherent break-in time of the polishing pads during CMP [54]. In comparison to Neat PU, the average MRR value of PUPCD40 (2.287 μm/h) exhibited a 14.5% increase, while showing a 4.2% improvement over PUPCD20 (2.190 μm/h), surpassing the commercial pad HF2 (1.732 μm/h) by 24.3% (shown in Figure 7b). This enhancement can be attributed to the improvements in surface hardness (shore D 58), wear index, bulk material of tensile strength, elongation at break, and density of the PUPCD40.

**Figure 8:**
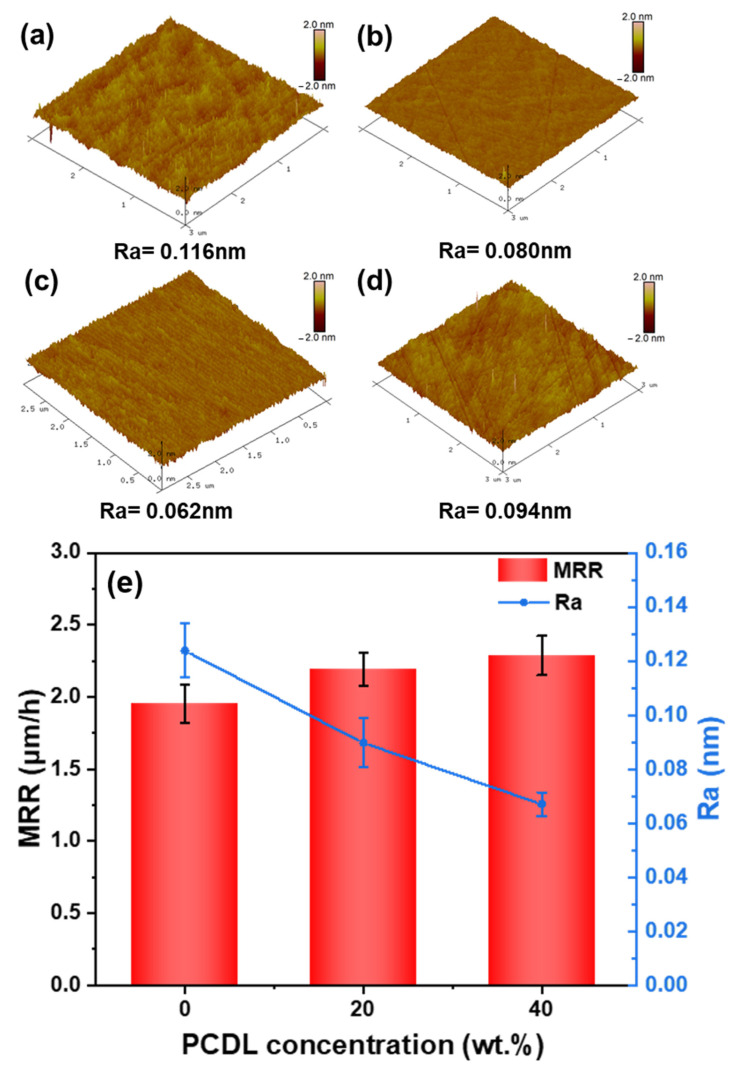
AFM images of the Si-polar surface center point of the SiC substrate with different PCDL contents: (**a**) Neat PU, (**b**) PUPCD20 (20 wt.%), (**c**) PUPCD40 (40 wt.%), (**d**) HF2 (commercial pad), and (**e**) variation diagram of average surface roughness and material removal rate with different PCDL contents.

Figure 8 illustrates the AFM images of the surface roughness (Ra) of the Si-face of SiC substrates following 12 h of CMP treatment with four different polishing pads, alongside the trends of MRR and Ra as a function of PCDL content. In Figure 8a–d, the surface roughness of the center point of the Si-face of SiC after 12 h of CMP with Neat PU, PUPCD20, PUPCD40, and HF2 polishing pads is depicted. Then, polishing SiC substrates with a KMnO_4_-based slurry containing MnO_2_ abrasives can result in a low latent scratch density in a shorter period compared to when using Al_2_O_3_ or SiO_2_ abrasives [21]. This indicates that employing the aforementioned slurry not only shortens processing time but also enhances substrate yield, ultimately contributing to a reduction in overall processing costs. Except for PUPCD40, noticeable scratches at picometer depth are observed on the Si face, with specific scratch depths detailed in Figure S2. When the PCDL content increases from 0 wt.% to 40 wt.%, there is a significant reduction in both the depth and quantity of scratches. Simultaneously, the MRR of the pad increases from 1.955 μm/h to 2.287 μm/h, and the average surface roughness of the Si-face of SiC decreases from 0.124 nm to 0.067 nm (as shown in Figure 8e). The AFM images reveal the presence of scratches on the SiC substrates, which are likely caused by abrasive particles trapped between the pad and the substrate during polishing [22]. The pad’s mechanical properties, such as hardness and elasticity, significantly influence scratch formation [17,55]. For instance, a harder pad may increase the pressure on abrasive particles, leading to deeper scratches, whereas a more elastic pad could mitigate scratch formation by absorbing mechanical stress. Further optimization of the pad’s properties, such as reducing hardness or enhancing elasticity, may help minimize scratch formation and improve surface quality.

To highlight the advantage of the PCDL-modified polyurethane pad with the KMnO_4_-based slurry for SiC substrate polishing, a summary of previously reported SiC polishing is presented in Table 3. In comparison to the materials in other studies, the PUPCD40 pad presents better CMP performance with the KMnO_4_-based slurry.

Compared with the surface roughness level of rough polishing achieved by the polyurethane polishing pad in the published literature, it is also necessary to carry out the fine polishing of the damping cloth polishing pad to meet the application requirements of SiC [56]. However, the one-step polishing of PUPCD40 samples in this study has achieved a fine polished surface roughness for SiC.

CMP is a process characterized by the friction-induced controlled abrasion of the SiC surface, mediated by the interaction between the pad and abrasives within a defined chemical environment of slurry. The above observation demonstrates that the addition of PCDL in a sample can obviously enhance the MRR and surface quality of the 4H-SiC substrate by reinforcing the mechanical and frictional properties of the bulk material, including hardness, density, elastic modulus, wear resistance, tensile strength, and elongation at break. To further elucidate the mechanisms underlying this intriguing phenomenon during CMP polishing, the discussion will be structured around two key aspects. Firstly, an oxidation reaction occurs between the KMnO_4_-based slurry and the surface of the SiC substrate (Equations (3) and (4)) [21]. The resultant oxide layer is then effectively removed through mechanical abrasion, facilitated by the MnO_2_ abrasives present on the polishing pad.

(3) $$M_{n}O_{4}^{-}\to M_{n}O_{2}\left(a\right)+O_{2}^{-}$$

(4) $${3O}_{2}^{-}+2SiC\to 2CO\left(g\right)+SiO_{2}+e^{-}$$

Conversely, the interaction between the polyurethane pad surface and the KMnO_4_-based slurry may result in a reaction where permanganate ions attack the ether bonds within the polyurethane structure, leading to chain scission and the generation of small molecules and oxidation products. The oxidation of ester bonds contributes to the degradation of polyurethane, resulting in the formation of carboxylic acids and alcohols. This degradation adversely affects the pore structure on the pad surface during the CMP process, consequently diminishing both the polishing efficiency and the lifetime of the pad. In contrast, the incorporation of PCDL-modified polyurethane polishing pads significantly improves the polishing rate while simultaneously achieving a high-quality surface finish on the SiC substrate.

## 4. Conclusions

In summary, we successfully prepared TDI-based polyurethane polishing pads modified with PCDL for polishing SiC substrates in strong oxidizing KMnO_4_-based slurry containing MnO_2_ abrasives. The results demonstrated that as the content of PCDL increased, both the mechanical properties and chemical corrosion resistance of the polishing pads improved significantly. The incorporation of PCDL polyol altered the ratio of soft segments to hard segments, leading to an increase in the glass transition temperature and a reduction in the degree of phase separation. When the PCDL loading reached 40 wt.% in the pad, notable enhancements were observed as follows: a 2.1-fold increase in tensile strength, a 4.2-fold increase in elastic modulus, a 1.6-fold increase in elongation at break, and a 0.5-fold decrease in wear index. Furthermore, the MRR of PUPCD40 improved to 2.287 μm/h, and the average Ra of SiC decreased from 0.124 nm to 0.067 nm, indicating improved polishing performance under these conditions. This research confirms that the strategic addition of PCDL to polyurethane formulations can significantly enhance the performance characteristics of CMP pads, particularly when used in aggressive environments such as those involving high concentrations of KMnO_4_. It also offers a feasible solution for the mass production of high-efficient polishing pads in that the processing time can be shortened, and the SiC substrate yield can be improved. Both contribute to reducing the process cost by one-step polishing.

## Figures and Tables

**Table 1:** Process parameters of SiC substrate polishing using a polisher.

Process Conditions	The First Step	The Second Step
Platen rotation speed (rpm)	80	80
Head rotation speed (rpm)	40	40
Polishing down force (g/cm^2^)	180	220
Slurry	H_2_O	COPOL-431 (MnO_2_, pH = 2.7)
Slurry flow rate (mL/min)	600	400
Conditioning/Polishing time (min)	15	120
SiC piece size (inch)/number (pcs)	/	6/4

**Table 2:** Physical properties of the porous polishing pad.

Parameter	Neat PU	PUPCD20	PUPCD40	HF2
Density (g/cm^3^)	0.695 ± 0.022	0.751 ± 0.020	0.828 ± 0.017	0.804 ± 0.023
Hardness (Shore D)	48 ± 2	50 ± 1	58 ± 1	53 ± 2
Compressibility (%)	1.51 ± 0.30	1.27± 0.20	0.78 ± 0.10	0.66 ± 0.20
Resilience (%)	57.7 ± 6.0	73.70 ± 3.1	87.29 ± 2.9	66.5 ± 6.3
Porosity (%)	41.62 ± 4.63	35.36 ± 3.52	30.13 ± 3.03	30.51 ± 4.06
Average pore size (μm)	118.2 ± 37.2	95.0 ± 34.3	68.3 ± 24.0	75.6 ± 19.2
Wear index (*I*)	125.8 ± 2.6	83.3 ± 2.1	60.3 ± 2.0	99.6 ± 3.1
Tensile strength (MPa)	8.199 ± 0.146	11.942 ± 0.474	17.171 ± 1.172	6.328 ± 0.355
Elasticity modulus (MPa)	17.098 ± 2.974	32.875 ± 4.497	71.140 ± 5.077	46.030 ± 2.411
Elongation at break (%)	120.61 ± 10.85	156.97 ± 17.99	187.40 ± 9.96	135.79 ± 5.87

**Table 3:** Comparison of MRR, Ra, and the process parameters of SiC polishing with previous publications.

Pad	Slurry	Rotation Speed (rpm)	Down Force (g/cm^2^)	Wafer Type	Si Face MRR (μm/h)	Ra (nm)	[Ref.]
IC-1000	KMnO_4_, MnO_2_ abrasive	40	300	4H-SiC	1.020	0.138	[21]
SUBA800	6.5 wt.% KMnO_4_, Al_2_O_3_ abrasive	100	422	4H-SiC	1.400	0.105	[56]
SUBA600	KMnO_4_, Al_2_O_3_, MnO_2_ abrasive	50	366	4H-SiC	1.300	0.210	[57]
IC-1400	0.1 wt.% KMnO_4_,SiO_2_ abrasive	100	212	4H-SiC	0.510	1.000	[58]
IC-1400	KMnO_4_, MnO_2_ abrasive	90	187	4H-SiC	0.600	1.01	[59]
IC-1000	KMnO_4_, SiO_2_/CeO_2_ abrasive	80	246	6H-SiC	1.207	0.216	[23]
PUPCD40	5 wt.% KMnO_4_, MnO_2_ abrasive	80	220	4H-SiC	2.287	0.067	This work

## Data Availability

The original contributions presented in this study are included in the article/Supplementary Materials. Further inquiries can be directed to the corresponding author (Yiming Meng).

## Supplementary Materials

The following are available online at https://www.mdpi.com/article/10.3390/polym17050613/s1, Figure S1: DSC curves of PU pads prepared under different PCDL1000 contents. Figure S2: Polishing surface topographies and depth of scratches on center point of SiC substrate by (a) Neat PU, (b) PUPCD20, (c) PUPCD40, and (d) HF2 pad CMP process after CMP process. Table S1: Formulation of samples. References [60,61,62,63,64] are cited in the Supplementary Materials.

## References

[1] Chen X., Yang X., Xie X., Peng Y., Xiao L., Shao C., Li H., Hu X., Xu X. (2023). Research progress of large size SiC single crystal materials and devices. Light Sci. Appl..

[2] Müller S.G., Glass R.C., Hobgood H.M., Tsvetkov V.F., Brady M., Henshall D., Jenny J.R., Malta D., Carter C.H. (2000). The status of SiC bulk growth from an industrial point of view. J. Cryst. Growth.

[3] Yasseen A.A., Zorman C.A., Mehregany M. (1999). Roughness reduction of 3C-SiC surfaces using SiC-based mechanical polishing slurries. J. Electrochem. Soc..

[4] Aida H., Doi T., Takeda H., Katakura H., Kim S.-W., Koyama K., Yamazaki T., Uneda M. (2012). Ultraprecision CMP for sapphire, GaN, and SiC for advanced optoelectronics materials. Curr. Appl. Phys..

[5] Tsai M.Y., Wang S.M., Tsai C.C., Yeh T.S. (2015). Investigation of increased removal rate during polishing of single-crystal silicon carbide. Int. J. Adv. Manuf. Technol..

[6] Musolino M., Carria E., Crippa D., Preti S., Azadmand M., Mauceri M., Isacson M., Calabretta M., Messina A. (2023). Development of n-type epitaxial growth on 200 mm 4H-SiC wafers for the next generation of power devices. Microelectron. Eng..

[7] Chen Z., Huang A.Q. (2024). Extreme high efficiency enabled by silicon carbide (SiC) power devices. Mater. Sci. Semicond. Process..

[8] Nakamura D., Gunjishima I., Yamaguchi S., Ito T., Okamoto A., Kondo H., Onda S., Takatori K. (2004). Ultrahigh-quality silicon carbide single crystals. Nature.

[9] Selder M., Kadinski L., Durst F., Hofmann D. (2001). Global modeling of the SiC sublimation growth process: Prediction of thermoelastic stress and control of growth conditions. J. Cryst. Growth.

[10] Choi I., Jeong H.Y., Shin H., Kang G., Byun M., Kim H., Chitu A.M., Im J.S., Ruoff R.S., Choi S.-Y. (2016). Laser-induced phase separation of silicon carbide. Nat. Commun..

[11] Hwang S., Park J., Kim W. (2024). The Stability Evaluation of Ceria Slurry Using Polymer Dispersants with Varying Contents for Chemical Mechanical Polishing Process. Polymers.

[12] Wang W., Lu X., Wu X., Zhang Y., Wang R., Yang D., Pi X. (2023). Chemical–Mechanical Polishing of 4H Silicon Carbide Wafers. Adv. Mater. Interfaces.

[13] Uneda M., Fujii K. (2020). Highly efficient chemical mechanical polishing method for SiC substrates using enhanced slurry containing bubbles of ozone gas. Precis. Eng..

[14] Prasad A., Remsen E.E., Xiang H. (2007). Surface chemical characteristics of CMP polyurethane pads. MRS Proc..

[15] Park K.H., Kim H.J., Chang O.M., Jeong H.D. (2007). Effects of pad properties on material removal in chemical mechanical polishing. J. Mater. Process. Technol..

[16] Tso P.-L., Hsu R. (2007). Estimating chemical mechanical polishing pad wear with compressibility. Int. J. Adv. Manuf. Technol..

[17] Kim B.S., Tucker M.H., Kelchner J.D., Beaudoin S.P. (2008). Study on the mechanical properties of CMP pads. IEEE Trans. Semicond. Manuf..

[18] Roy P.K., Deopura M., Misra S. (2010). Customized Polishing Pads for CMP and Methods of Fabrication and Use Thereof.

[19] Chiu W.-L., Huang C.-I. (2023). Polymer Nanoparticles Applied in the CMP (Chemical Mechanical Polishing) Process of Chip Wafers for Defect Improvement and Polishing Removal Rate Response. Polymers.

[20] Huy L.N.Q., Lin C.-Y., Chen C.-C.A. (2022). Development of modeling to investigate polyurethane pad hardness in chemical mechanical planarization/polishing (CMP) process. Jpn. J. Appl. Phys..

[21] Nakamura T., Kumagai A., Saruwatari Y., Hara S. (2023). Improving the polishing speed and surface quality of 4H-SiC wafers with an MnO_2_- based slurry. Solid State Phenom..

[22] Yu S., Hu J.J., Xu L.L., Liu M., Liu E., Givens J., Leighton J. (2022). Highest quality and repeatability for single wafer 150mm SiC CMP designed for high volume manufacturing. Mater. Sci. Forum.

[23] Ni Z., Chen G., Xu L., Zhang P., Dai M., Qian S., Bian D., Zhang H. (2021). Preparation of ceria-coated silica nanoparticles and their chemical mechanical planarization performance on Si-face 6H-SiC substrates. ECS J. Solid State Sci. Technol..

[24] Iwata T., Tsurumaki A., Tajima S., Ohno H. (2014). Fixation of ionic liquids into polyether-based polyurethane films to maintain long-term antistatic properties. Polymer.

[25] Cakić S.M., Ristić I.S., Krakovský I., Stojiljković D.T., Bělský P., Kollová L. (2014). Crystallization and thermal properties in waterborne polyurethane elastomers: Influence of mixed soft segment block. Mater. Chem. Phys..

[26] Rogulska M., Kultys A., Podkościelny W. (2007). Studies on thermoplastic polyurethanes based on new diphenylethane-derivative diols. II. Synthesis and characterization of segmented polyurethanes from HDI and MDI. Eur. Polym. J..

[27] Wang L.F., Ji Q., Glass T.E., Ward T.C., McGrath J.E., Muggli M., Burns G., Sorathia U. (2000). Synthesis and characterization of organosiloxane modified segmented polyether polyurethanes. Polymer.

[28] Leng Y., Zhang Y., Chen X., Yi C., Fan B., Wu Q. (2011). Hydrophobic thermoplastic starches modified with polyester-based polyurethane microparticles: Effects of various diisocyanates. Ind. Eng. Chem. Res..

[29] Yildirim E., Yurtsever M. (2014). The role of diisocyanate and soft segment on the intersegmental interactions in urethane and urea based segmented copolymers: A DFT study. Comput. Theor. Chem..

[30] Jewrajka S.K., Kang J., Erdodi G., Kennedy J.P., Yilgor E., Yilgor I. (2009). Polyisobutylene-based polyurethanes. II. Polyureas containing mixed PIB/PTMO soft segments. J. Polym. Sci. Part A Polym. Chem..

[31] Erdodi G., Kang J., Kennedy J.P., Yilgor E., Yilgor I. (2009). Polyisobutylene-based polyurethanes. III. Polyurethanes containing PIB/PTMO soft co-segments. J. Polym. Sci. Part A Polym. Chem..

[32] (2021). Standard Test Methods for Vulcanized Rubber and Thermoplastic Elastomers-Tension.

[33] (2019). Standard Test Method for Abrasion Resistance of Organic Coatings by the Taber Abraser.

[34] Naderizadeh S., Athanassiou A., Bayer I.S. (2018). Interfacing superhydrophobic silica nanoparticle films with graphene and thermoplastic polyurethane for wear/abrasion resistance. J. Colloid Interface Sci..

[35] Liu C., Yin Q., Yuan Q., Hao L., Shi L., Bao Y., Lyu B., Ma J. (2022). A wear-resistant, self-healing and recyclable multifunctional waterborne polyurethane coating with mechanical tunability based on hydrogen bonding and an aromatic disulfide structure. Polym. Chem..

[36] Badri K.B.H., Sien W.C., Shahrom M., Hao L.C., Baderuliksan N.Y., Norzali N. (2010). FTIR spectroscopy analysis of the prepolymerization of palm-based polyurethane. Solid State Sci. Technol.

[37] Tanaka T., Yokoyama T., Yamaguchi Y. (1968). Quantitative study on hydrogen bonding between urethane compound and ethers by infrared spectroscopy. J. Polym. Sci. Part A-1 Polym. Chem..

[38] Yılgör E., Yılgör İ., Yurtsever E. (2002). Hydrogen bonding and polyurethane morphology. I. Quantum mechanical calculations of hydrogen bond energies and vibrational spectroscopy of model compounds. Polymer.

[39] Lee H.S., Wang Y.K., Hsu S.L. (1987). Spectroscopic analysis of phase separation behavior of model polyurethanes. Macromolecules.

[40] Barbeau P., Gerard J.F., Magny B., Pascault J.P. (2000). Effect of the diisocyanate on the structure and properties of polyurethane acrylate prepolymers. J. Polym. Sci. Part B Polym. Phys..

[41] He Y., Zhang X., Zhang X., Huang H., Chang J., Chen H. (2012). Structural investigations of toluene diisocyanate (TDI) and trimethylolpropane (TMP)-based polyurethane prepolymer. J. Ind. Eng. Chem..

[42] Piril Ertem S., Yilgor E., Kosak C., Wilkes G.L., Zhang M., Yilgor I. (2012). Effect of soft segment molecular weight on tensile properties of poly(propylene oxide) based polyurethaneureas. Polymer.

[43] Hu J., Mo R., Sheng X., Zhang X. (2020). A self-healing polyurethane elastomer with excellent mechanical properties based on phase-locked dynamic imine bonds. Polym. Chem..

[44] Sáenz-Pérez M., Lizundia E., Laza J.M., García-Barrasa J., Vilas J.L., León L.M. (2016). Methylene diphenyl diisocyanate (MDI) and toluene diisocyanate (TDI) based polyurethanes: Thermal, shape-memory and mechanical behavior. RSC Adv..

[45] He Y., Xie D., Zhang X. (2014). The structure, microphase-separated morphology, and property of polyurethanes and polyureas. J. Mater. Sci..

[46] Eceiza A., Martin M.D., de la Caba K., Kortaberria G., Gabilondo N., Corcuera M.A., Mondragon I. (2008). Thermoplastic polyurethane elastomers based on polycarbonate diols with different soft segment molecular weight and chemical structure: Mechanical and thermal properties. Polym. Eng. Sci..

[47] Park K., Oh J., Jeong H. (2008). Pad Characterization and Experimental Analysis of Pad Wear Effect on Material Removal Uniformity in Chemical Mechanical Polishing. Jpn. J. Appl. Phys..

[48] Lee C.-F., Chen C.-W., Rwei S.-P., Chuang F.-S. (2021). Thermal behavior and morphology of thermoplastic polyurethane derived from different chain extenders of 1,3- and 1,4-butanediol. Appl. Sci..

[49] Bajaj R., Desai M., Jairath R., Stell M., Tolles R. (1994). Effect of polishing pad material properties on chemical mechanical polishing (CMP) processes. MRS Online Proc. Libr. (OPL).

[50] Huang H., Pang H., Huang J., Yu P., Li J., Lu M., Liao B. (2021). Influence of hard segment content and soft segment length on the microphase structure and mechanical performance of polyurethane-based polymer concrete. Constr. Build. Mater..

[51] Ostanin S.A., Mokeev M.V., Pikhurov D.V., Sakhatskii A.S., Zuev V.V. (2022). Interplay of structural factors in molecular dynamics of microphase-separated or microphase-mixed structures of polyurethanes revealed by solid-state NMR and dielectric spectroscopy. Chem. Phys. Impact.

[52] Liu B., Tian H., Zhu L. (2015). Structures and properties of polycarbonate modified polyether-polyurethanes prepared by transurethane polycondensation. J. Appl. Polym. Sci..

[53] Eceiza A., Larrañaga M., de la Caba K., Kortaberria G., Marieta C., Corcuera M.A., Mondragon I. (2008). Structure–property relationships of thermoplastic polyurethane elastomers based on polycarbonate diols. J. Appl. Polym. Sci..

[54] Jeong H.D., Park K.H., Cho K.K. (2007). CMP pad break-in time reduction in silicon wafer polishing. CIRP Ann..

[55] Kenchappa N., Popuri R., Chockkalingam A., Jawali P., Jayanath S., Redfield D., Bajaj R. (2021). Soft chemical mechanical polishing pad for oxide CMP applications. ECS J. Solid State Sci. Technol..

[56] Wang W., Liu W., Song Z. (2021). Two-step chemical mechanical polishing of 4H-SiC (0001) wafer. ECS J. Solid State Sci. Technol..

[57] Cui D., Zhang B., Xian W., Liu M., Wu J., Liu S., Qin S., Wang Y., Liu Y. (2024). Unveiling the synergistic interaction: Investigating the enhanced mechanism of 4H–SiC chemical mechanical polishing with the addition of sodium silicate and manganese dioxide. Mater. Sci. Semicond. Process..

[58] Yin T., Doi T., Kurokawa S., Ohnishi O., Yamazaki T., Wang Z.D., Tan Z. (2012). The effects of strong oxidizing slurry and processing atmosphere on double-sided CMP of SiC wafer. Adv. Mater. Res..

[59] Yin T., Doi T., Kurokawa S., Zhou Z.z., Feng K.p. (2018). Polishing characteristics of MnO_2_ polishing slurry on the Si-face of SiC wafer. Int. J. Precis. Eng. Manuf..

[60] Pang J., Zhong J., Pu Z., Yang K., Yang Y., Yue M., Wu L. (2024). Study on synthesis of polycarbonate dilate polyurethane elastomers. J. Polym. Res..

[61] Wang X., Yang Z., Zhang Y., Wang T., Li S., Wang Q., Zhang X. (2024). Syncretic of soft, hard, and rigid segments cultivate high-performance elastomer. Chem. Eng. J..

[62] Gama N.V., Ferreira A., Barros-Timmons A. (2018). Polyurethane Foams: Past, Present, and Future. Materials.

[63] Dodge J. (2003). Polyurethanes and Polyureas. Synthetic Methods in Step-Growth Polymers.

[64] Jiao L., Xiao H., Wang Q., Sun J. (2013). Thermal degradation characteristics of rigid polyurethane foam and the volatile products analysis with TG-FTIR-MS. Polym. Degrad. Stab..

