# Supplementary material for: Effect of Structurally Modified Toluene Diisocyanate-Based Polyurethane Pads on Chemical Mechanical Polishing of 4H Silicon Carbide Substrate
Source: Polymers (Basel). 2025 Feb 25;17(5):613. doi: 10.3390/polym17050613 (PMC11902601; doi:10.3390/polym17050613)
Supplement: Supplementary file 1 [file polymers-17-00613-s001.zip › polymers-3474385-supplementary.pdf]

Supplementary Materials

Effect of Structurally Modified Toluene Diisocyanate-based Polyurethane Pad on Chemical Mechanical Polishing of 4H Silicon Carbide Substrate

Table S1. Formulation of samples.

Samples	Prepolymer (phr)[1]	PCDL (phr)[1]	L-1500 (phr)[1]	A33 (phr)[1]	DIW (phr)[1]	MOCA (phr)[1]
Neat PU	100	0	1.6	0.10	0.09	26
PUPCD20	100	30	2.0	0.12	0.08	15
PUPCD40	100	80	2.5	0.15	0.07	11

[1]phr: parts per hundred in the prepolymer, DIW: deionized water.

The formulation design of PUPCD40 and other samples were primarily determined by the content of reactive isocyanate groups (-NCO) that the ratio of isocyanate group to hydroxyl groups or amine groups (R value) in the polyurethane prepolymer [1-3]. In this study, the -NCO groups in the prepolymer were consumed by the hydroxyl groups in PCDL, the amine groups in MOCA, and the hydroxyl groups in deionized water. This consumption allowed for the calculation of the corresponding ratios used in the formulations.[4].

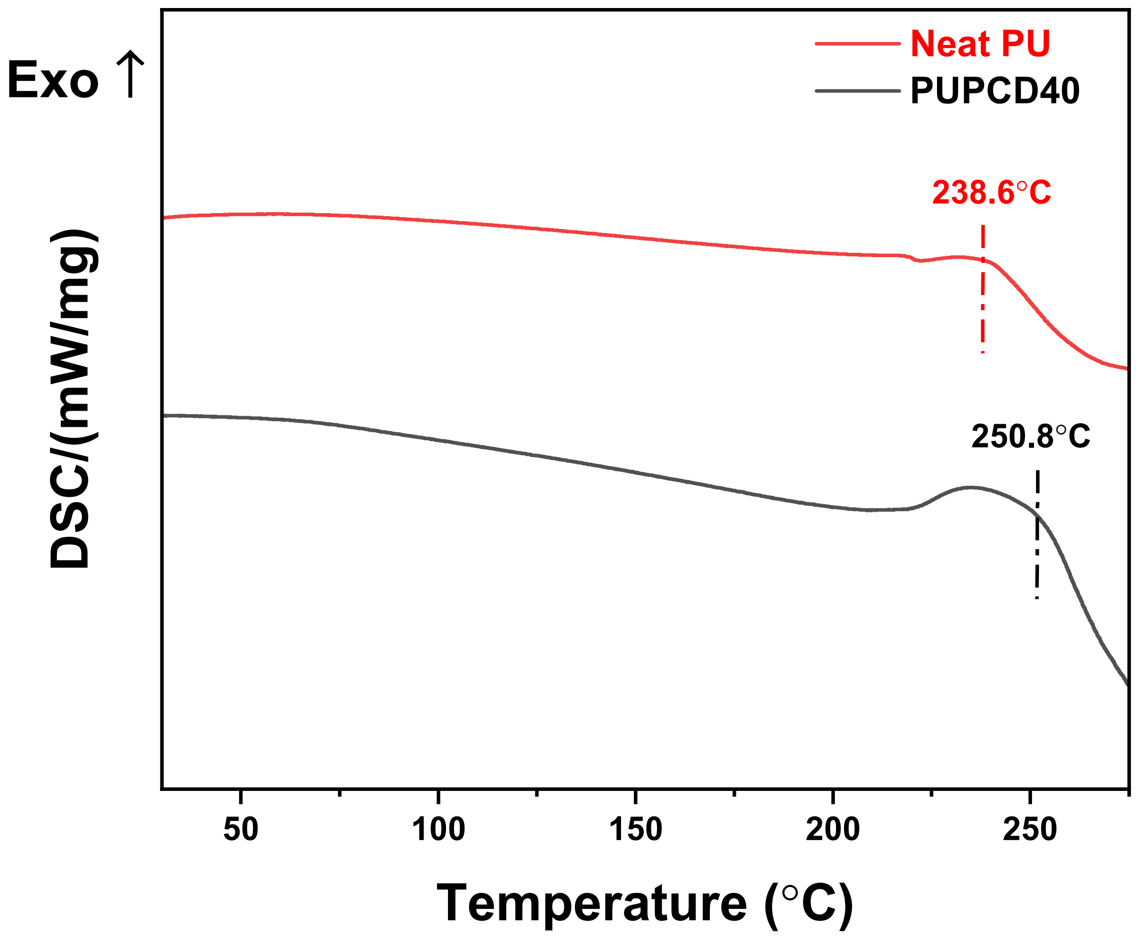

Figure S1. DSC curves of PU pads prepared under different PCDL1000 content.

DSC curves of the polishing pads, showing the glass transition temperature (T_g) for each sample. The T_g of PUPCD40 (250.8°C) is determined from the midpoint of the transition region. The initial rise in the PUPCD40 curve reflects thermal relaxation and segmental mobility before the glass transition, while the decline corresponds to the transition itself [5].

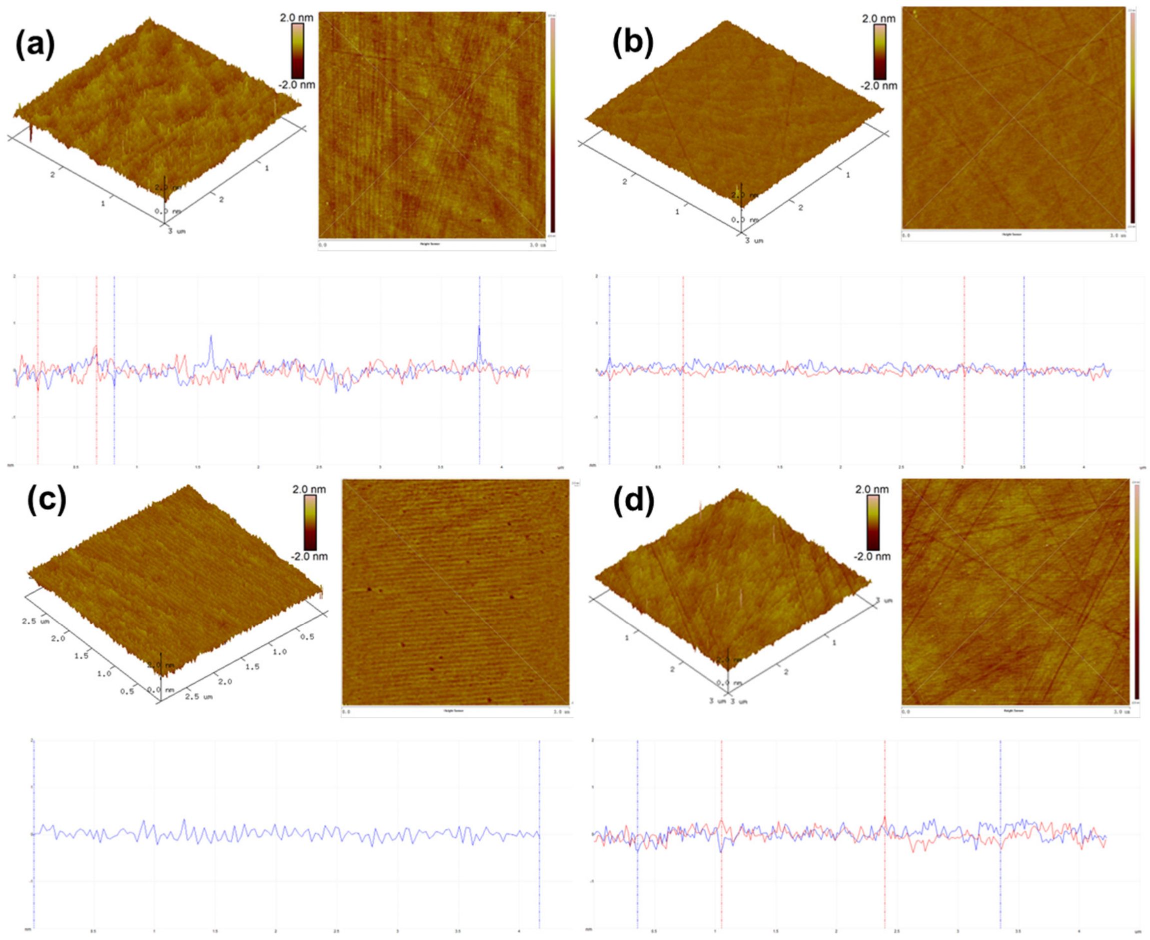

Figure S2. Polishing surface topographies and depth of scratches on center point of SiC substrate by (a) Neat PU, (b)PUPCD20, (c) PUPCD40, and (d) HF2 pad CMP process after CMP process.

References

1. Pang, J.; Zhong, J.; Pu, Z.; Yang, K.; Yang, Y.; Yue, M.; Wu, L. Study on synthesis of polycarbonate dilate polyurethane elastomers. *Journal of Polymer Research* **2024**, *31*, 149.
2. Wang, X.; Yang, Z.; Zhang, Y.; Wang, T.; Li, S.; Wang, Q.; Zhang, X. Syncretic of soft, hard, and rigid segments cultivate high-performance elastomer. *Chem. Eng. J.* **2024**, *495*, 153466.
3. Gama, N.V.; Ferreira, A.; Barros-Timmons, A. Polyurethane Foams: Past, Present, and Future. *Materials* **2018**, *11*, 1841.
4. Dodge, J. Polyurethanes and Polyureas. In *Synthetic Methods in Step‐Growth Polymers*; 2003; pp. 197-263.
5. Jiao, L.; Xiao, H.; Wang, Q.; Sun, J. Thermal degradation characteristics of rigid polyurethane foam and the volatile products analysis with TG-FTIR-MS. *Polymer Degradation and Stability* **2013**, *98*, 2687-2696.
